# Long term changes in the quality of the aquatic environment of thermally polluted Lake Licheńskie, Central Poland

**DOI:** 10.1016/j.ejrh.2025.102917

**Published:** 2025-12

**Authors:** Michał Woszczyk, Michael Brechbühler

**Affiliations:** a Biogeochemistry Research Group, Adam Mickiewicz University, Bogumiła Krygowskiego 10, Poznań 61-680, Poland; b Department of Life Safety and Environmental Protection, School of Earth Sciences, D. Serikbayev East Kazakhstan Technical University, Serikbayeva 19, Öskemen 070000, Kazakhstan; c Department Surface Waters Research & Management, EAWAG, Überlandstrasse 133, Dübendorf 8600, Switzerland

**Keywords:** Lake, Thermal pollution, Salinization, Alkalinity, Water quality

## Abstract

**Study region:**

Central Europe, Gniezno Lake District, Poland

**Study focus:**

The purpose of this study is to depict and explain long-term trends in surface water temperatures (LSWT) and chemical composition in Lake Licheńskie (LLi), which since 1960s has been involved in a cooling system of two electric power plants (PP) and thus has been prone to thermal pollution (TP). For the analyses we used 24-year long stationary monitoring and satellite data.

**New hydrological insights for the region:**

We estimated that LLi was 3.81°C warmer than natural lakes in the region and demonstrated that the TP displayed spatial and seasonal variability. The data shows that owing to a reduction in the PP activity the LSWT has constantly been decreasing at a rate of 0.09°C·y^−1^. Because, the lake has also been supplied with saltwater and highly alkaline effluents from nearby brown coal mine, LLi has been prone to salinization and alkalinization. The former process is still ongoing but alkalinization is declining, which is interpreted as a self-recovery of the lake triggered by a reduction in brown coal mining in the region. The knowledge of environmental conditions in the lake as well as its long-term changes is crucial for developing lake management strategies in the face of planned incorporation of the lake in a cooling system of the new nuclear power plant in the vicinity of the lake.

## Highlights

• Lake Licheńskie is thermally polluted by cooling water discharges from the power plant.
• The surface water temperature in Lake Licheńskie is 3.81°C higher than in non-heated lakes.
• The thermal pollution shows a decreasing trend.
• Lake Licheńskie is prone to long-term salinization and eutrophication but shows decline in alkalinity.

## Graphical Abstract

**Figure:**
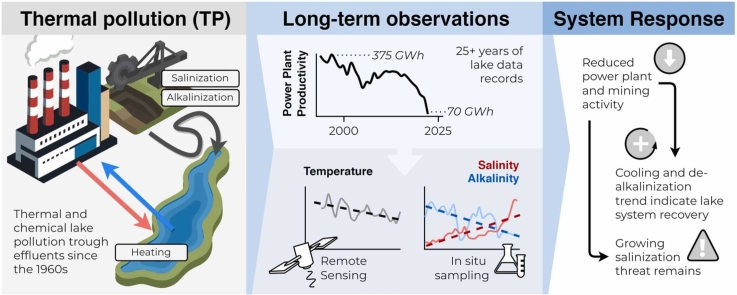

## 1 Introduction

Thermal pollution (TP) involves an increase (or decrease) in temperature of aquatic systems to non-natural levels and thus leads to a distortion in thermal equilibrium between the systems and the ambient atmosphere. Because pollution with cold water is rather uncommon (Kennedy, 2004), the TP is usually tantamount to the excess warming of lakes and/or rivers. The warming is primarily driven by a point-source discharges of heated industrial effluents, from energy-producing industry (both coal-based and nuclear) in particular (Rosen et al., 2015, Raptis et al., 2016, Miara et al., 2018, Yavari and Qaderi, 2020; Li et al.l, 2025), with some, usually minor, contribution from other processes (e.g. deforestation, urban runoff; Dodds and Whiles, 2010 and references therein). The highest discharges of heated waters are released by the power plants working in an once-through mode, in which the water collected from a river/lake is passed through the heat exchangers and finally rejected back into its source albeit at temperature 8 – 12°C higher (Madden et al., 2013, Raptis et al., 2016). As of the US for example it is estimated that nearly 50 % of surface waters thereof is exposed to thermal pollution (Echols et al., 2009). Rivers seem to be more jeopardized to TP than the other aquatic systems because they often receive direct inputs of heated waters. Raptis et al. (2016) showed that owing to this problem some rivers (e.g. Mississippi, Rhine, Po, Weser, Danube, Schelde) are permanently or temporarily heated over very long stretches of their valleys with the estimated warming of up to 12°C. In lakes the thermal pollution is much less recognized but the available data indicate that the surface water temperature increase due to discharges of cooling waters is in the order of 1 – 2 °C in Lake Stechlin (Germany; Kirillin et al., 2013), 0.3 °C in Lake Bienne (Switzerland; Vinnå et al. 2017), 2 – 6 °C in lakes Clinton, Newton and Coffeen (US; Mulhollem et al., 2016), 1.2 °C in Urias Lagoon (Mexico; Cardoso-Mohedano et al., 2015) and 0.5 – 6.3 °C in Lake Macquarie (Australia; Ingleton and Minn 2012).

Thermal pollution possesses considerable environmental threats to the affected rivers/lakes because of its multifaceted negative impacts on hydrodynamics, nutrient cycles, water quality and biota (Davidson and Bradshow, 1967; Ingleton and McMinn, 2012; Kirillin et al., 2013; Madden et al., 2013; Cardoso-Mohedano et al., 2015; Raptis et al., 2017; Vinnå et al. 2017; Dziuba et al., 2020; Gashkina and Moiseenko, 2020). In lakes TP is thought to stabilize vertical stratification and consequently leads to deterioration of red-ox conditions in the near bottom water. Given that the energy demand is increasing globally and conventional energy sources are still gaining importance, the TP and its consequences in aquatic systems will likely remain a concern-rising issue in the following decades.

Despite the fact that the thermally polluted lakes can act as test sites for the research on some potential consequences of climate warming in aquatic systems and resilience of the latter to the temperature changes, the lakes have not been extensively studied so far. The current study provides a comprehensive analysis of Lake Licheńskie, the most heavily thermally polluted lake in Poland.

**Fig. 1:**
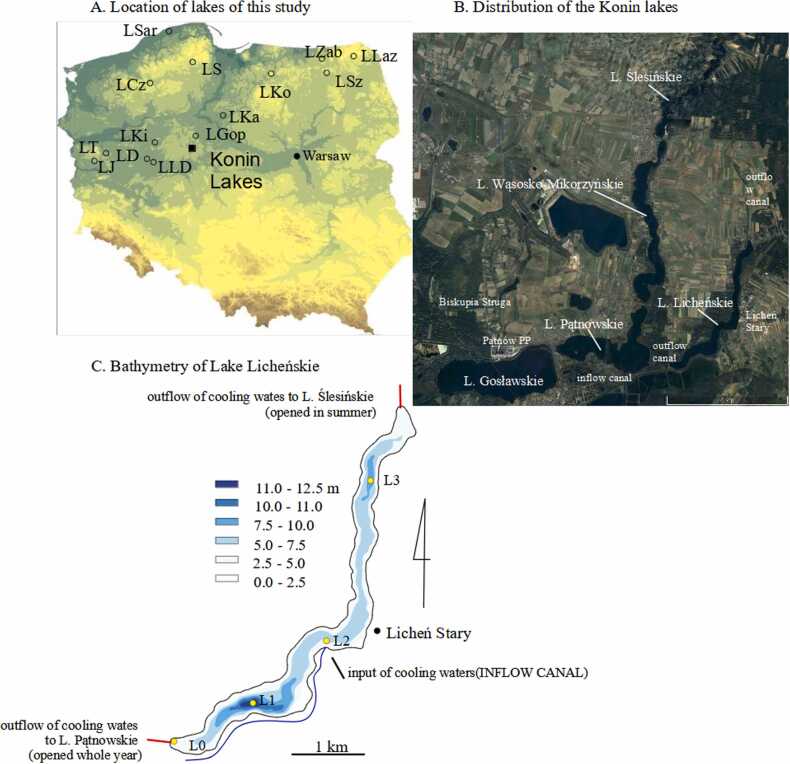
Location and bathymetry of Lake Licheńskie. A) Location of L. Licheńskie and other lakes mentioned in the text. B) The lakes involved in the cooling system of the KPPP. C) Bathymetric map of L. Licheńskie and location of the data collection sites. LCz - L. Czarne, LD – L. Dębno, LGop - L. Gopło, LJ - L. Jasne, LKa - L. Kamionkowskie, LKi - L. Kierskie, LKo - L. Kortowskie, LLaz - L. Łazduny, LLD – L. Łódzko-Dymaczewskie, LS - L. Suminko, LSar - L. Sarbsko, LSz – L. Szurpiły, LT - L. Trześniowskie, LZab - L. Żabińskie.

Lake Licheńskie (LLi, central Poland), together with a few adjacent lakes (Lake Gosławskie, Lake Pątnowskie, Lake Wąsosko-Mikorzyńskie and Lake Ślesińskie), for over 50 years has been involved in the cooling system of the Konin – Pątnów power plants (KPPP). The hydrographic network in the vicinity of the KPPP has been modified so that the power plants could use water from the lakes for cooling electricity generators. Naturally existing lakes were connected (or reconnected) by a system of canals to enforce constant water circulation over a distance of 32 km, sufficiently long to ensure undisturbed supply of cold water to the power plants. To further enhance the heat transfer to the atmosphere, the cooling system is equipped with a few pumping stations, spillways and weirs localized between the connected lakes. The KPPP collect water from Lake Gosławskie and Lake Pątnowskie (Fig. 1) and discharge it to the canals, which distribute the effluents over the whole system of connected lakes. The disposal of cooling effluents has considerably transformed the Konin lakes. In case of LLi the discharge led to the increase in surface water temperatures (LSWT), reduction of spatial extent and duration of winter ice as well as disturbances in seasonal vertical water mixing and changes in chemical composition of water. Some authors claimed that the lake had shifted from dimictic regime towards monomixis (Socha and Hutorowicz, 2009, Stawecki et al., 2013) while others postulated that it had become polymictic (Brzozowska et al., 2007). In addition, owing to continuous throughflow of cooling water, the lake has a very high horizontal water exchange rate (water renewal time) which is currently estimated to 2 – 9 days (Stawecki et al., 2013). In addition, the emissions of CH_4_ and N_2_O from the lake to the atmosphere are considerably low (Woszczyk and Schubert, 2023). The latter observation acts as an interesting aspect of the transformation of the system because anthropogenic impact usually leads to enhancement of GHG production in lakes. Last but not least, the TP of LLi has also facilitated the spread of invasive plant and animal species (Zdanowski et al., 2020) and enforced water organisms to develop adjustment strategies to living in a heated habitat (Dziuba et al., 2020). Owing to the above changes, LLi is found to be the most heavily human-altered lake in the system of the Konin lakes.

Despite that LLi has been intensely used for economical purposes for decades, in the following years the human pressure on the lake is expected to increase because of the plans for setting up a 2.8 GW nuclear power plant (NPP) in Pątnów. The NPPs are known to be more environmentally impactful than the coal-fired plants (Raptis et al., 2017). This new NPP will succeed the present day brown coal-fired Pątnów PP (PPP) and will also use the system of Konin lakes as a source of cooling water. Therefore, before any management decisions regarding the lake are made, it is reasonable to assess the degree of transformation of this system and to identify possible environmental threats to the lake (if there are any) that may arise during further exploitation.

In the current study we combined existing historical monitoring records of lake water parameters (collected between 1995 and 2023), spaceborne thermal images (from 2000 to 2025) and our own hydrochemical data from LLi to investigate long-term changes to the lake. Based on this we found that, unlike in other Polish lakes, during the last three decades the lake surface water temperatures in LLi have shown a decreasing trend. There was also a considerable increase in lake water salinity and a decline in alkalinity. From the data it thus follows that, to large degree, the lake processes have followed a different trajectory than in natural lakes prone to ongoing environmental changes related to global warming. At the same time, the changes to the lake can act as a manifestation of processes of adaptation of the system to declining anthropogenic pressure.

Our results demonstrate that despite being heavily disrupted, lakes display considerable capacity to re-equilibrate with the ambient environment. We believe, that this study will contribute to better understanding of the functioning of aquatic systems under a strong human pressure but also will be helpful in developing strategies for sustainable management and protection of lakes.

## 2 Study area

Lake Licheńskie (LLi) is a small (1.48 km^2^ surface area; 7.47 ×10^6^ m^3^ water volume) and shallow (mean water depth 4.5 m; max depth 12.5 m) post-glacial lake located in the SE part of the Gniezno Lake District, central Poland (Fig. 1A). The lake is classified as moderately eutrophic (Pyka et al., 2007) and is known to be home for some invasive and thermophilus plants and fish such as *Vallisneria spiralis* and *Pseudorasbora parva*, respectively. LLi acts as the smallest in the system of the Konin lakes encompassing also Lake Gosławskie (LGo; 4.54 km^2^; 13.8 ×10^6^ m^3^; 5 m max depth), Lake Pątnowskie (LPa; 2.82 km^2^; 7.26 ×10^6^ m^3^; 5.5 m max depth), Lake Wąsosko-Mikorzyńskie (LWM; 2.53 km^2^; 29.3 ×10^6^ m^3^; 36.5 m max depth) and Lake Ślesińskie (LSl; 1.52 km^2^; 11.6 ×10^6^ m^3^) (Fig. 1B). The lake catchment is primarily occupied by agriculture (60 %) and forests (22 %). Urban areas constitute c.a. 9 % of the catchment.

Since 1960s the lakes have been involved in the cooling system of the Konin and Pątnów electric power plants (KPPP). The cooling waters from the Pątnów PP (PPP) are discharged to LGo and then, via the system of canals, flow to LLi and LWM. The temperatures of these waters vary between 13 and 15°C in winter and 30°C in summer (Fig. S1) and are on average 8.2 ± 2.8°C warmer than at the entrance to the cooling system. On its way to LLi, the PPP effluents meet the Konin PP (KPP) cooling waters. The inflow of cooling waters to LLi is from the south and the mouth of the inflow canal is in the middle part of the lake (Fig. 1C). Over the long term the discharge of the KPPP effluents received by LLi has declined and currently it is estimated to c.a. 22 m^3^·s^−1^. From LLi, the cooling waters, after 2 – 9 day-long retention, outflow to LPa (outflow 1; Fig. 1C) and/or to LSl (outflow 2; Fig. 1C). The former outflow is active throughout the whole year, while the latter is only open during summers.

The water level in the Konin lakes is artificially kept constant at 84.90 – 85.10 m asl in LGo, 83.70 – 84.10 m asl in LLi and between 83.17 and 83.87 m asl in LPa, LWM and LSl. During dry periods the system of Konin lakes is supplied with water from the Warta river. However, there is also whole-year-round water input from the natural, albeit, anthropogenically transformed tributaries, the Struga Kleczewska and the Struga Biskupia (SB), in particular. These streams receive discharges of post-mining waters from the nearby brown coal mines and thus are important for shaping chemical composition of the Konin lakes.

## 3 Methods

The study is based on self-collected hydrochemical observations, archival limnological data and satellite-based thermal data.

### 3.1 LLi water sampling and in situ measurements

LLi water samples were taken monthly in three sites, L1, L2 and L3 (Fig. 1B), between March 2022 and February 2023. Locations of these sites were chosen so that they covered spatial variability of thermal pollution in the lake. L1 is located in the deepest point (12.5 m) in the SW part of the lake, L2 is located just in front of the mouth of the discharge canal and L3 is located in 8-m deep depression in the north part of the lake. The L1 site was previously monitored in 2014/15 (Woszczyk and Schubert, 2023). In all these sites we measured lake water temperature, conductivity, pH, and dissolved O_2_. The measurements were performed from the surface to the bottom with 1-m resolution using YSI Professional Plus probe, calibrated and checked with certified reference material (Harbour water, NWHAMIL-20.2) beforehand. The lake surface water was sampled for total P (P_tot_) and alkalinity (A_t_) and the samples were taken to PTFE containers from just below air-water interface.

### 3.2 Acquisition of archival limnological data

For sake of comparison with the LLi data we also used multi-parameter records from several lakes in Poland (Fig. 1A). The data was taken from both open- and closed-access databases. Albeit the data was collected in different periods, there was considerable overlapping of the datasets with monitoring of LLi.

Historical limnological data from the Konin Lakes for 1995 – 2023 was obtained at the Department of Environmental Protection of the Zespół Elektrowni Pątnów-Adamów-Konin, Poland (ZEPAK; https://www.zepak.com.pl/en). The ZEPAK has monitored the physical-chemical parameters of the lakes since the opening of the KPPP in late 1960s. The observations (e.g. surface water temperatures, LSWT [°C]; conductivity, κ [μS·cm^−1^]; pH; alkalinity, A_T_ [mmol·L^−1^]; calcium, Ca^2+^ [mg·L^−1^]; chlorides, Cl^-^ [mg·L^−1^]; chemical oxygen demand, COD [mgO_2_·L^−1^]; total P, P_tot_ [mg·L^−1^]) were collected at L0 station (Fig. 1B) from surface waters with monthly resolution and using standard ISO methods (pH: PN-EN ISO 10523:2012; κ: PN:EN 27888:1999; A_T_: PN-EN ISO 9963–1:2001 +Ap1:2004; Ca^2+^: PN-ISO 6058:1999; COD: PN-EN ISO 8457:2001; P_tot_: PN-EN ISO 6878:2006 +Ap1:2010 +Ap2:2010 pt 7; Cl^-^: PN-EN ISO 10304–1:2009 +AC:2012).

The data from L. Suminko (LS), L. Szurpiły (LSz) and L. Łazduny (LLaz) encompassing monthly records of SWT and A_T_ for the period between October 2007 and May 2010 was obtained on request from prof. Wojciech Tylmann (University of Gdańsk, PL). A_T_ data from L. Kierskie (LK) was obtained from prof. Karina Apolinarska (Adam Mickiewicz University, Poznań, PL) (Apolinarska et al., 2020). The data on Cl^-^ in L. Żabińskie (LZab) for 2012 – 2022 is available at https://doi.org/10.34808/bsk4-eg58 (Tylmann et al., 2023). Monitoring data on A_T_ and Cl^-^ from L. Czarne (LCz), L. Kamionkowskie (LKa) and L. Kortowskie (LKo) was obtained on request at the Polish Chief Inspectorate of Environmental Protection (GIOŚ). The former two lakes are involved in the Integrated Environmental Monitoring (ZMŚP) programme of the GIOŚ. Hydrochemical data from L. Łódzko-Dymaczewskie (LLD), L. Dębno (LD) and L. Trześniowskie are available at https://doi.pangaea.de/10.1594/PANGAEA.977483.

### 3.3 Collection of satellite-based thermal data

Satellite-derived thermal data from LLi and adjacent lakes were obtained from the Landsat Level 2 Collection 2 (L2C2) archive (U.S. Geological Survey, 2020), covering the period from January 2000 to January 2025. Surface temperature products, derived through standard atmospheric correction procedures, were used directly. Initial scene selection was based on cloud coverage, retaining only images with less than 75 % total cloud cover. Subsequently, a minimum threshold of 10 % clear-sky water coverage over the study area was applied, resulting in a dataset of 693 thermal images acquired by the Landsat 7 Enhanced Thematic Mapper Plus (ETM+) (Earth Resources Observation and Science EROS Center, 2020a), Landsat 8 Thermal Infrared Sensor (TIRS), and Landsat 9 Thermal Infrared Sensor 2 (TIRS-2) (Earth Resources Observation and Science EROS Center, 2020b) processed data at 30 m spatial resolution, as provided in the L2C2 products. This resolution results from resampling coarser native thermal data (60 m for ETM+; 100 m for TIRS and TIRS-2) and is appropriate given the spatial scale of the study lakes, allowing meaningful analysis of intra-lake thermal variability. Satellite overpasses occurred at a mean acquisition time of 09:30 UTC ± 20 min (1σ), corresponding to approximately 10:30 or 11:30 local time depending on the application of daylight saving time.

Cloud masking was performed using CFMask (Foga et al., 2017) quality assessment flags, retaining only clear-sky water pixels. Pixels flagged as ice or exhibiting surface temperatures below 0 °C were assigned a value of 0 °C to account for frozen surface conditions. Where multiple acquisitions were available for the same day, mosaics were generated by prioritizing images with greater clear-sky water pixel coverage.

The agreement between satellite-based estimates of LSWT and ground observation was assessed using same-day match-ups with available in situ measurements collected by local authorities and corresponding satellite observations. For each sampling site, satellite-based temperatures were extracted as the mean of a 3 × 3 pixel window (90 ×90 m) centered on the measurement location.

To address data gaps and irregular temporal sampling, we employed a gap-filling approach combining lake-specific seasonal-trend decomposition and Empirical Orthogonal Function (EOF) analysis. Prior to decomposition, outlier filtering was applied using a Z-score threshold (|Z| > 2.5) to remove residual land and cloud-contaminated pixels. For each lake, the corresponding time series was then decomposed into seasonal, trend, and residual components using Seasonal-Trend decomposition based on LOESS (STL; Cleveland et al., 1990), which extracts smoothed estimates of each component using a locally estimated scatterplot smoothing (LOESS) method based on local polynomial regressions. Spatial gaps in the residual component were addressed using the Data Interpolating Empirical Orthogonal Functions (DINEOF) method (Beckers and Rixen, 2003, Alvera-Azcárate et al., 2005), followed by linear temporal interpolation to address temporal gaps. The gap-free residuals were then recombined with the seasonal and trend components to reconstruct the complete, continuous daily lake surface water temperature (LSWT) time series for each lake.

Reconstruction uncertainty was assessed using internal cross-validation within the DINEOF algorithm, applied to the residual component of the STL-decomposed temperature series. For each lake, 1 % of valid water pixels were randomly withheld, reconstructed, and compared with their original values.

### 3.4 Analytical methods

Chemical composition of water was analyzed on the day of collection or the following day the latest. P_tot_ was analyzed with cuvette photometric tests LCK349 by Hach-Lange. Unfiltered samples were first mineralized using LT200 thermostat (Hach-Lange) and then analyzed with DR 1900 spectrophotometer (Hach-Lange). The average recovery of P measurements, assessed on the basis of certified reference materials, was 90 ± 3 % (m ± SD). Total alkalinity A_T_ was determined by titration with 0.1 M HCl with regard to methyl orange. The average recovery of A_T_ analyses was 105 ± 2 % (m ± SD).

### 3.5 Calculations and statistical data treatment

Stratification stability index (W_S_; J·m^−2^) (Idso, 1973) acts as a measure of stability of vertical stratification of lake water column and is calculated as

(1) $${\text{W}}_{\text{s}}=\frac{\text{g}}{{\text{A}}_{\text{l}}}\int_{0}^{{\text{Z}}_{\text{m}}}{(\text{z}-\text{z}}^{*}{)(\text{ρ}}_{\text{z}}{-\text{ρ}}_{\text{mean}}{)\text{A}}_{\text{z}}\text{dz}$$

where g represents acceleration due to gravity (m·s^−2^), A_l_ – lake surface area (m^2^), A_z_ – surface area at depth *z* (m^2^), z – depth (m), z* - depth at ρ (m), ρ_z_ – water density at z (kg·m^−3^) and ρ_mean_ – mean lake water density in the water column (kg·m^−3^). Lake water density ρ_z_ was calculated as a function of temperature, salinity and hydrostatic pressure according to Sun et al. (2008). The higher the W_S_, the stronger is the vertical thermal (and density) stratification of lake waters.

Rates of removal of O_2_ from the near bottom waters (mmol·m^−2^·d^−1^) were calculated from the slope of the linear regression line (mmol·d^−1^) relating the changes of the near-bottom water O_2_ over time to the area of the near bottom water (m^−2^) (Matthews et al., 2005).

Saturation index for calcite SI_calc_ was calculated from activities of aCa^2+^ [mol·kg^−1^] and aCO_3_^2-^ [mol·kg^−1^] and solubility product K_calc_ with the formula

(2) SI_calc_ = (aCa^2+^·aHCO_3_^-^)/K_calc_

The ion activities were obtained as described by Woszczyk et al. (2023) i.e. using ionic strength derived from measured conductivity κ.

Statistical data treatment involved calculating Pearson’s correlation coefficients *r* (and corresponding probability values *p*) between variables, assessing statistical significance of the differences obtained with the Kruskall-Wallis test supported with Dunn’s post hoc test as well as determining statistical significance of temporal trends with Mann-Kendall test. The calculations were carried out with Past 4.09 (Hammer et al., 2001).

## 4 Results

### 4.1 LSWT in Lake Licheńskie and adjacent lakes

To compare the temperature regime of the Konin lakes as well as to assess the differences between the latter group and the natural lakes in the area we used LSWT values extracted from the spaceborne thermal images from 2000 — 2025 instead of long-term in-situ instrumental records collected at L0 site (Fig. 1B) between 1995 and 2024. The major advantage of this approach was that it used spatially resolved temperature data for each lake which, given the considerable areal LSWT variability in the Konin lakes, enabled us to avoid poor representativeness of a single site-based monitoring for the whole lake systems. The validation based on same-day comparisons with in situ measurements yielded mean absolute errors (MAE) of 0.36 °C, 0.39 °C, and 0.44 °C for LGo, LLi, and LSl, respectively, with corresponding standard deviations of 0.41 °C to 0.56 °C (Fig. S2, Tab. S1). These errors are notably low given that the match-ups were uncoordinated and therefore subject to uncertainties arising from differences in observation time of day (i.e., diurnal cycle), skin effect, georeferencing accuracy, and measurement conditions such as sampling depth.The internal cross-validation indicates that the DINEOF-based LSWT gap reconstruction performs consistently across all lakes. Mean absolute error (MAE) values range from 0.35 ± 0.41 °C to 0.44 ± 0.56 °C (±1σ), reflecting low average gap reconstruction uncertainty in the LSWT fields (Tab. S2).

**Fig. 2:**
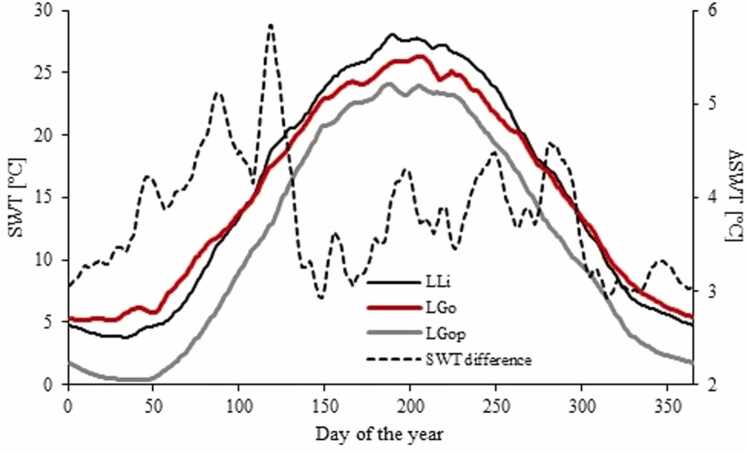
Annual SWT cycle in L. Licheńskie (LLi; black), L. Gosławskie (LGo; red) and L. Gopło (LGop; grey) as well as SWT differences (ΔSWT) between LLi and LGop calculated on the basis of surface averaged temperature values for the period between 2000 and 2025. LLi – L. Licheńskie, LGo – L. Gosławskie, LSl – L. Ślesińskie, LWM – L. Wąsosko-Mikorzyńskie, LPat – L. Pątnowskie.

This analysis showed that the mean daily LSWT in LLi during the last two and a half decade varied between 3.8 and 28.0°C and that these values were statistically different from those in other Konin lakes, except LGo (Fig. S3; Tab. S2). At the same time, the LSWT differences between LGo and LPa as well as LWM were insignificant. Although thermally similar overall, LLi and LGo exhibited seasonal differences in LSWT patterns. During spring and summer (mid-April to mid-October) the LSWT values in LLi were up to 2.7°C higher than in LGo, while from mid-October to mid-April the opposite was true (SWT in LGo was up to 2.1°C warmer than in LLi) (Fig. 2). Except for LSl, the LSWTs in the Konin lakes for the period 2000 – 2025 were significantly higher than in L. Gopło, acting as a non-heated natural reference lake. The LSWT differences between LLi and LGop showed quite irregular annual pattern and ranged from 2.9 to 5.8°C (on average 3.8°C; Fig. 2).

**Fig. 3:**
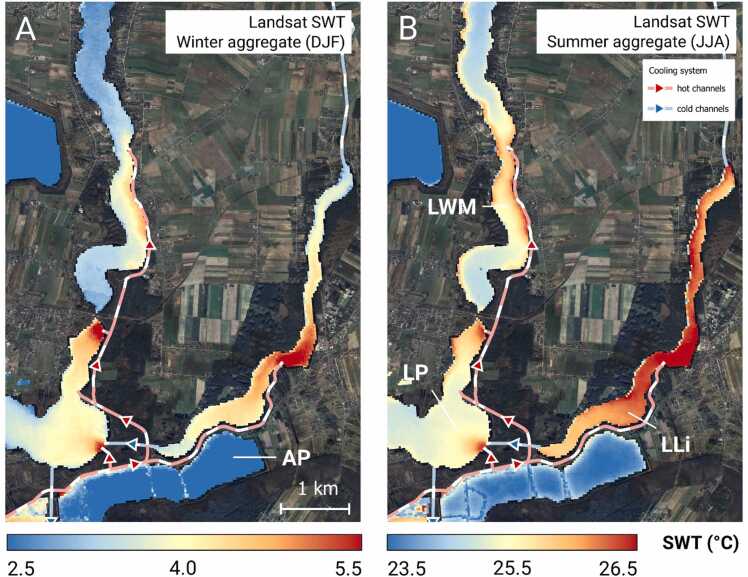
Long-term spatial SWT distribution in L. Licheńskie and adjacent lakes in winter (DJF) and summer (JJA).

The spatial distribution of LSWT in LLi varied seasonally. As shown in Fig. 3, throughout the whole year the highest SWT values occurred adjacent to the mouth of the inflow canal (the L2 site). The differences in the mean daily LSWT between the L2 site and L1 and L3 stations, situated outside the plume, varied between 0.5 and 2.8°C and were statistically significant. However, in winter (DJF) the spatial temperature gradients were stronger than in the summer (JJA) and the ΔSWT between L2 and L1/L3 sites were 1.82—2.78°C ($\overset{®}{x}$=2.37°C) and 1.58—2.56°C ($\overset{®}{x}$=2.16°C), respectively. In summer (JJA), the lake was more thermally homogenous with respective ΔLSWT values from 0.79—1.74°C ($\overset{®}{x}$=1.30°C) and 0.53—1.39°C ($\overset{®}{x}$=0.93°C). Despite the fact that the LSWTs in L1 and L3 were not significantly different, for the major part of the year the north part of LLi (L3 site) was warmer than the west part of the lake (L1 site) and the average ΔLSWT was 0.26 ± 0.29 °C.

Considerable role in protecting the lake from overheating was played by the outflow to Lake Ślesińskie (outflow 2; Fig. 1C). The weir in the north part of LLi, directing the water to LSl, opened whenever the LSWT reached 30°C thus leading to a rapid drop in temperature of up to 8°C within a month (Fig. S4).

The distribution of temperature in the lake water column showed that LLi was prone to a density stratification. Stratification build-up was between April and May and the break-up occurred between August and September, however in 2023 a weak stratification in the near bottom waters persisted until November (Fig. S5). The stratification stability index (W_s_) values for LLi during the maximum stability reached 86.7 and 96.9 J·m^−2^ in 2014 and 2023 respectively. In L1 and L3 sites the stratification built up synchronously and a thermocline (Δ temperature/Δ depth of up to 7°C/1 m) occurred between 1 and 3 m depth. In L2 the stratification developed more irregularly. There was no continuous period of summer stratification. Instead, stratification was observed occasionally in March and November, when the water column in L1 and L3 was homothermic. The thermocline was weak (up to 2.5°C/1 m) and often occurred close to the lake surface (between 0 and 1 m depth).

**Fig. 4:**
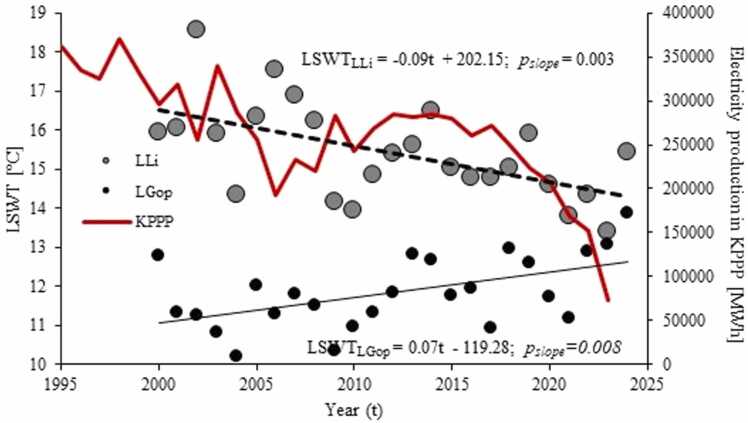
Long-term SWT trends in L. Licheńskie (grey circles) and L. Gopło (black circles) between 2000 and 2025 as well as annual energy production in the KPPP between 1995 and 2023. The circles represent annual mean SWT for the lakes.

Over the long term, the LSWT in LLi displayed a statistically significant declining trend and the rate of change was −0.09°C·y^−1^ (*p* = 0.003) (Fig. 4). Significant tendencies were also obtained in LGo (-0.10°C·y^−1^; *p* = 0.008) and LPa (-0.07°C·y^−1^; *p* = 0.007), while the SWT in LWM and LSl remained unchanged (Fig. S6). At same time in LGop there occurred an increasing temperature trend at 0.07°C·y^−1^ (*p* = 0.008) (Fig. 4).

### 4.2 Hydrochemistry of Lake Licheńskie and adjacent lakes: present day conditions and long-term changes

The mean annual A_T_ in LLi between 1995 and 2023 ranged from 4.1 to 5.2 mmol·L^−1^ (Fig. S7). These values were significantly different from LGo and LSl but not from LWM and LPa (Tab. S3). The measurements conducted in 2022–23 in three sites in LLi showed only minor differences in A_T_ throughout the lake. A_T_ in LLi varied in an annual cycle between maximum in winter (January – March) and the lowest values in August and September (Fig. S8).

**Fig. 5:**
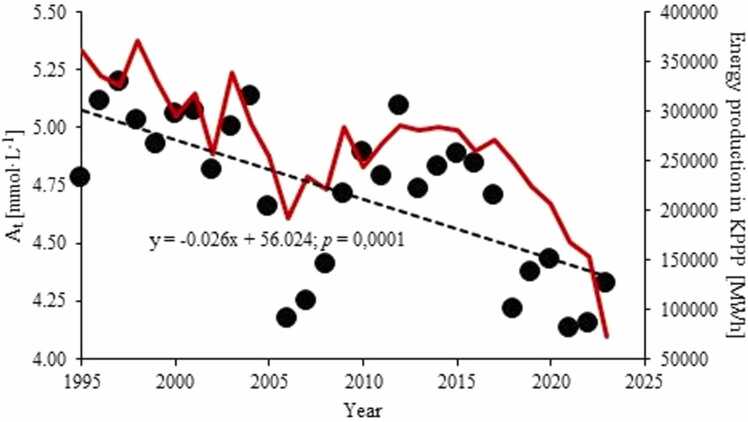
Long term A_T_ trends in Lake Licheńskie between 1995 and 2023. The electricity production in the KPPP (in red) shown for comparison.

Over the long-term, A_T_ concentrations in all Konin lakes have displayed marked statistically significant declining trends (Fig. 5; Fig. S9). In LLi the A_T_ has been decreasing at a rate of 26 µmol·L^−1^·y^−1^. In LMW, LSl and LPa the rates were similar, while in LGo the decrease in A_T_ pool was as high as 42 µmol·L^−1^·y^−1^.

The mean annual pH of the LLi waters was between 7.07 and 8.46 and these values were not significantly different from other Konin lakes (Fig. S10). In an annual cycle there was a maximum in spring while the lowest values occurred in October (Fig. S11). Since the mid-1990s the mean annual pH values in all Konin lakes have remained unchanged. The long-term median pH was 8.40.

Throughout the last 25 years LLi surface waters have constantly showed highly positive SI_calc_ values of 1.2 ± 0.5 ($\overset{®}{x}\pm \sigma$) implying persistent supersaturation with CaCO_3_ in the epilimnion. The highest SI_calc_ were in spring-early summer and the lowest in autumn (Fig. S12). The long term trend in SI_calc_ was statistically insignificant.

Mean annual values of chemical oxygen demand (COD), acting as a proxy for the concentration of O_2_-consuming chemical species (primarily organic matter) in lake water, varied between 5.4 and 8.9 mg O_2_·L^−1^ (Fig. S13) and the differences between LLi and other Konin lakes were minor and statistically insignificant. The COD displayed a slight long-term increase, however the trend was not statistically significant.

**Fig. 6:**
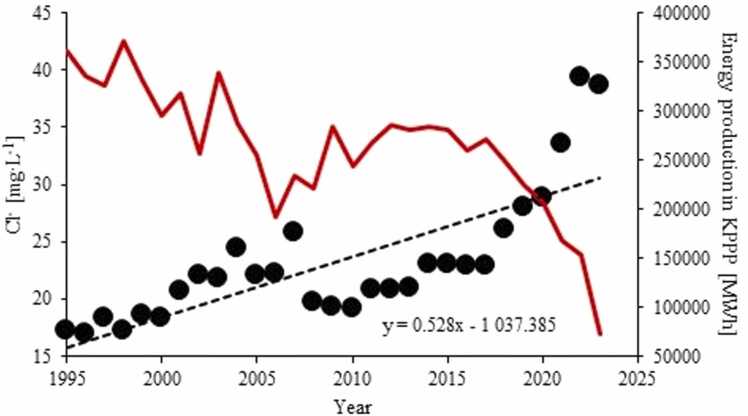
Long term Cl^-^ trend in Lake Licheńskie between 1995 and 2023. The electricity production in the KPPP (in red) shown for comparison.

Mean annual Cl^-^ concentrations in LLi waters for the period from 1995 to 2023 varying between 16.9 and 39.4 mg·L^−1^ were similar to the Cl^-^ throughout the Konin lakes (15.9 – 40.3 mg·L^−1^) (Fig. S14). The Cl^-^ showed only minor and insignificant annual variability. In long-term perspective, the Cl^-^ concentrations in LLi displayed a significant increasing trend at a rate of 0.53 mgCl^-^·L^−1^·y^−1^ (Fig. 6). The trend was stepwise, however, and in a recent decade, the Cl^-^ increase has clearly accelerated. Compared to the LLi water chlorinity in the late 1990s (1995 – 2000), the Cl^-^ concentrations has doubled in a time span of 20 years. The same tendencies were identified in other Konin lakes (Fig. S15).

**Fig. 7:**
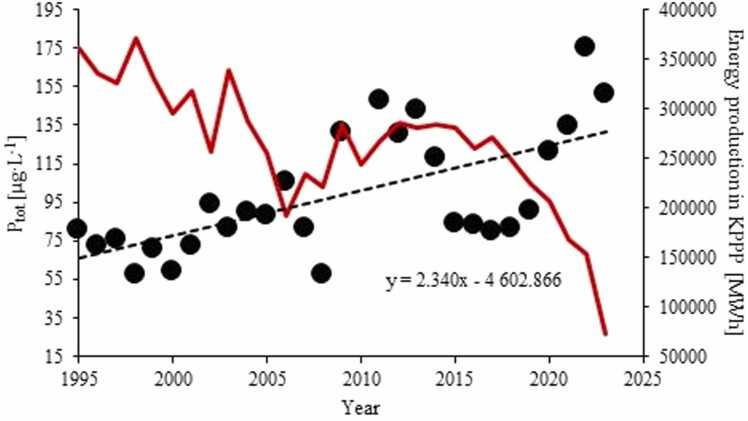
Long term P_tot_ trend in Lake Licheńskie between 1995 and 2023. The electricity production in the KPPP (in red) shown for comparison.

Total phosphorous (P_tot_) concentrations in LLi varied between 58 and 175 µg·L^−1^ which fitted well the range of 38 – 182 µg·L^−1^ obtained in other Konin lakes (Fig. S16). Based on data from 2022 to 23, there were no spatial differences in P_tot_ throughout the lake. On an annual basis, considerably higher P_tot_ values were during winters (DJF) while the lowest values typically occurred in June (Fig. S17). Between 1995 and 2023 the P_tot_ in LLi has increased substantially at an overall rate of 2.7 µg·L^−1^·y^−1^, albeit the increasing trend was non-linear (Fig. 7). The statistically significant increase occurred in March, May and October – November (Tab. S4).

## 5 Discussion

### 5.1 The problem of thermal pollution of Lake Licheńskie

Because LLi is one of the smallest lakes (in terms of volume) in the KPPP cooling system and since it has received a major part of thermal effluents discharged from the KPPP (30 % from the PPP and 54 % from KPPP; Stawecki et al., 2007) for over the last 60 years, it is regarded the most strongly thermally polluted in a group of Konin lakes. Hilbricht-Ilkowska and Zdanowski (1988) estimated long-term SWT increase in LLi for c.a. 5.2 – 12.9°C, which was considerably higher than 3 – 7°C warming in L. Gosławskie and L. Pątnowskie, 3 – 4°C in L. Ślesińskie and 1 – 2°C in L. Wąsosko-Mikorzyńskie. Our results are consistent with the above pattern, albeit with some differences. First, the thermal pollution of LLi and LGo was similar, and the best estimate for the lake area-averaged warming was 3.81°C and 3.64°C, respectively. These values appear high compared to the LSWT increase of 0.8°C and 0.23–0.32°C in L. Stechlin and L. Biel, respectively, both heated by the effluents discharging from the local NPPs (Kirillin et al., 2013, Vinnå et al., 2017). Second, LSl, which displayed only minor and insignificant LSWT difference to LGop (on average 1.30 ± 0.41°C), seemed to be close to its natural thermal regime. Three, LPa and LWM showed a moderate degree of thermal pollution and the LSWT difference between these lakes and non-heated waters was 2.23 ± 0.64°C and 2.33 ± 0.50°C, respectively.

In LLi, there was considerable spatial variability of thermal pollution from 5.30 ± 0.70°C in the plume zone to 3.70 ± 0.55°C – 3.44 ± 0.54°C in the north and west parts of the basin, respectively, and the warming varied irregularly throughout the year between 2.93 and 5.84°C. In LGo the seasonal changes were stronger than in LLi. The maximum thermal pollution (6.49°C) was in winter and the minimum (1.11°C) – in summer. In winter LGo was thus warmer than LLi while in summer the opposite situation occurred. The disparity between LGo and LLi in an annual course of thermal pollution arises from different distances between the lakes and the source of thermal effluents as well as different heat storing capacities of LGo and LLi. LGo is the very first recipient of cooling waters from the PPP (currently a major source of pollution) and it is where a large part of the heat delivered is lost. Under the simplifying assumption that the daily discharge of cooling waters (1.9 ×10^6^ m^3^) is spread over the whole lake surface (4.54 ×10^6^ m^2^), the time required to decrease the temperature of the effluents to the values measured in LGo water is approximately between 20 h in winter and 86 h in summer (Table 1). Thus, during the time of retention in LGo (38–173 h; Hilbricht-Ilkowska, Zdanowski, 1988) the thermal effluents are considerably cooled down, even to the temperatures of the ambient air. It is worth noting, that in winter the rate of cooling is higher than in summer, which agrees with the findings by Vinnå et al. (2017), and is explicable on the grounds of the Newton’s law of cooling on the proportionality between the heat loss and the water-air temperature gradient (Davidzon, 2012). During summer, different mechanisms of the heat transfer in the water columns of shallow (LGo) and deep lakes (LLi) come to the fore. Toffolon et al. (2022) demonstrate that shallow lakes tend to have higher thermal inertia compared to deep lakes, which results from the fact that in the former the heat absorbed is distributed throughout the whole lake water volume, and consequently the thermally effective layer encompass the whole water column. On the other hand, in deep lakes, the heat transfer is limited by the vertical stratification. Consequently, most of the heat delivered to such a lake is stored in relatively thin layer above the thermocline, which translates into the higher temperatures in the surface waters. This effect is likely to occur in LLi, which, in contrast to LGo, is prone to seasonal stratification (Woszczyk and Schubert, 2023), and can be invoked to explain the higher LSWT values than in LGo. Thermal pollution of lakes is often associated with the changes in vertical density stratification of lake water column. However, the effects of pollution on the hydrodynamic conditions in lakes can be different. Kirillin et al. (2013) reported on eliminating winter stratification and weakening summer stratification in L. Stechlin. At the same time these authors found that the duration of summer stratification increased owing to thermal discharges to the lake. Vinnå et al. (2017), on the other hand, showed that in L. Biel the stratification was enhanced both in terms of water column stability and duration. Via strengthening thermal gradient between epi- and hypolimnion, the thermal pollution vicariously led to deterioration of oxygen conditions in the near bottom water (Vinnå et al. 2017).

**Table 1:** Estimation of cooling time of KPPP thermal effluents in L. Gosławskie.

Month	h	c	ρ	T_0_	T_1_	T_env_	t_c_
	W·m^−2^·K^−1^	J·K^−1^·kg^−1^	kg·m^−3^	[°C]			[h]
January	4191	24.2	1000	13.0	5.2	0.6	20
July	4174	24.2	1000	31.1	25.87	25.80	86

The time of cooling *t*_*c*_ was calculated from the Newton’s law of cooling expressed with the formula: $t_{c}=\frac{\ln(\frac{T_{0}-T_{\mathrm{env}}}{T_{1}-T_{\mathrm{env}}})}{k}$, where T_0_ stands for the initial temperature of cooling waters, T_1_ is the final temperature of cooling waters (equal to the SWT in LGo) and T_env_ is the temperature of the ambient air. The coefficient *k* was derived as $k=h\frac{A_{\mathrm{lake}}}{c∙\rho ∙V_{\mathrm{water}}}$, where *A*_*lake*_ stands for lake surface, *V*_*wate*r_ is volume of thermal effluents discharging during one day, *c* stands for specific heat capacity, *h* is heat transfer coefficient and *ρ* is water density.

**Fig. 8:**
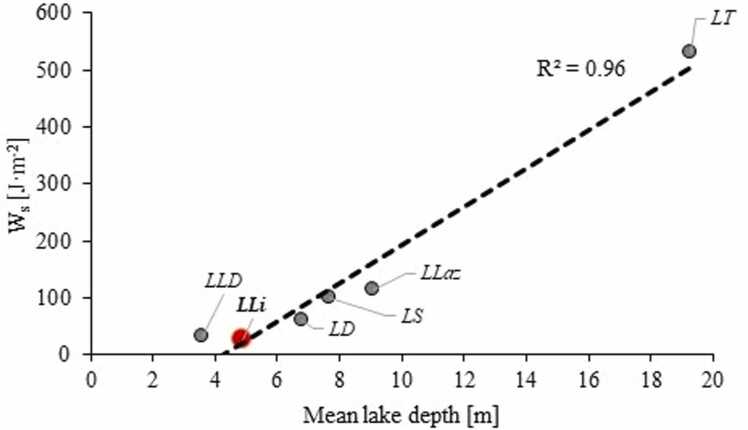
Stratification stability index W_s_ as a function of mean lake depth for selected Polish lakes. Location of the lakes is shown in Fig. 1.

The data from LLi were insufficient to assess the changes to the stratification stability and the length of the stratification in the lake over time. However, stratification stability index (Ws) for LLi was rather low and typical for the lake of its depth (Fig. 8). In fact, the values obtained in LLi were among the lowest of all lakes compared. It might thus indicate that the long-term disposal of thermal effluents has not translated into stronger vertical stability of LLi water column. Accordingly, the oxygen cycling in the lake was similar to natural lakes. The O_2_ accumulation rate in the near-bottom water layer of LLi was 0.5 mmol·m^−2^·d^−1^ while in LD and LLD (location shown in Fig. 1A), similar in terms of bathymetry and trophic conditions (Woszczyk and Schubert, 2023), 1.2 mmol·m^−2^·d^−1^ and 1.3 mmol·m^−2^·d^−1^ was obtained, respectively. The O_2_ consumption rates estimated to 1.4 mmol·m^−2^·d^−1^ in LLi, 1.5 mmol·m^−2^·d^−1^ in LD and 1.9 mmol·m^−2^·d^−1^ in LLD were even more similar (Fig. S18). From the data it thus appears that the thermal pollution has not affected the oxygenation of the water column.

Between 2000 and 2024 the mean annual LSWT in LLi showed a decreasing trend (Fig. 4). The cooling was also displayed by LGo and LPa, but not by LWM and LSl. The long-term temperature pattern contrasts with the tendencies observed in natural Polish lakes, which are prone to warming (Piccolroaz et al., 2021; Ptak et al., 2019, Ptak et al., 2020), such as it was recorded in L. Gopło. In the latter lake between 2000 and 2025 the LSWT has increased at 0.07°C·y^−1^. The discrepancy between the LSWT trends in the Konin lakes and natural lakes can presumably be attributed to the considerable reduction in the KPPP activity over time (Fig. 4). Since the first half of 2010s the production of electricity in the power plants has dropped by nearly 70 %. Given that a strong positive relationship exists between the operability of the KPPP and the temperature of the thermal effluents (r = 0.61 at *p* < 0.001), this would definitely translate into the decline in the capacity of heat delivered to the system of Konin lakes. The absence of any LSWT trend in LWM and LSl is consistent with a lower degree of thermal pollution in these lakes and results from compensating effect of climate-induced warming on the cooling due to KPPP decline.

## 6 Long-term changes in lake water quality

60 years of involvement in the KPPP cooling system led to considerable changes in chemical composition of LLi waters. Since the mid-1990s the lake surface water has displayed growing, statistically significant trends in chlorides and P_tot_ concentrations. At the same time there was a decreasing trend in alkalinity. The increase in Cl^-^ occurred at a rate of 0.5 mgCl^-^·L^−1^·y^−1^, which translated into ca 150 % increase within 28 years. Interestingly, the Cl^-^ trends in the lakes were inversely related to the KPPP operability (Fig. 6). The increase in Cl^-^ indicate that the system of Konin lakes has been prone to salinization, acting as an intensifying and expanding environmental issue on a global scale which is estimated to affect about one third of freshwater aquatic systems worldwide (Canedo-Argüelles 2020). From Polish lakes the data on salinization is scarce and show different patterns. For example, in L. Czarne (Fig. 1A) a decreasing trend emerges from Cl^-^ data for 1995 – 2023 and in Lake Kamionkowskie (Fig. 1A) the Cl^-^ concentrations between 2015 and 2023 have remained unchanged. On the other hand, however, 10 year-long monitoring of L. Żabińskie (Fig. 1A; Tylmann et al., 2023), a pristine lake in NE Poland, revealed a slight, albeit statistically significant (*p* = 0.036), increasing trend in concentrations of Cl^-^ (0.36 mgCl^-^·L^−1^·y^−1^) in this lake, thus providing evidence for ongoing salinization in spite of minor anthropogenic disturbance. Similarly, in Lake Kortowskie (Fig. 1A), a highly eutrophicated lake located in an urbanized area (the city of Olsztyn, N Poland), between 2007 and 2021 a statistically significant Cl^-^ increase at a rate of 0.44 mgCl^-^·L^−1^·y^−1^ can been found.

A number of drivers for salinization of freshwaters include different primarily anthropogenic factors such as direct salt deposition (e.g. by using road salt), resource extraction (e.g. through disposal of high salinity mining waters), agriculture (through the use of fertilizers and irrigation), forest clearing, urban and industrial wastewater discharges as well as human-induced leaching of natural and artificial landscapes (Kaushal et al., 2018; Canedo-Argüelles 2020; Thorslund et al., 2021). The salinization of LLi is presumably owing to the delivery of salt water drainage from nearby brown coal mines and/or wastewater treatment plants. These effluents are discharged to the Struga Biskupia (SB), which flows to Lake Gosławskie, the first lake in a cooling system, and then to other Konin lakes. By mixing of the SB water and LGo waters, the solutions are circulated throughout the whole cooling system. The link between salinization of LLi and disposal of mining and municipal wastewaters is corroborated by i) highly positive correlation between Cl^-^ in SB waters with those in LLi (r = 0.62 significant at *p* < 0.001) and ii) synchronicity of the Cl^-^ changes throughout the whole cooling system between 1995 and 2023. At the same time, the concentrations of Cl^-^ in LLi were higher than in SB (23.2 ± 5.8 mg·L^−1^ in LLi vs 17.7 ± 7.7 mg·L^−1^ in SB; the difference significant at *p* = 5.6·10^−23^). The difference can presumably be attributed to retention of salt in connected lakes, acting as a semi-closed system. Salts delivered by the SB circulate between the lakes in the cooling system and only during high water levels the excess water is dropped outside, to the Warta river. On the other hand, given that the SB is not exposed to thermal pollution, the Cl^-^ difference between LLi and SB can result from evaporative enrichment of the LLi waters.

Ecological effects of salinization of freshwater systems have thoroughly been described (Herbert et al., 2015). The most severe threats are associated with rapid salinity increases such as that in 2022 in the Odra river (W Poland/E Germany). During this event, along the major part of the river course there occurred massive fish mortality (Sługocki and Czerniawski, 2023).

At the same time, in spite of the very high nutrient level in LLi, since 1995 we have observed still ongoing increase in trophic state of this lake (Fig. 7). The P_tot_ values are currently above 100 μg·L^−1^, indicating hypetrophic conditions in the lake. A strong increase in eutrophication of LLi contrasts to a general tendency to reduce nutrient export from river watersheds across Poland and an observed tendency to improve trophic quality of lake waters in Poland (Neverova-Dziopak, 2021). There are three explanations for the high fertility of LLi. First, the enhanced P_tot_ values may be associated with phosphorus export from primarily agricultural catchment of LLi, as demonstrated by Zawiska et al. (2023). Second, delivery of nutrients by the mining waters discharging to the Konin lakes can contribute to the elevated P_tot_ in lake. The latter hypothesis can be drawn from a positive correlation of P_tot_ concentrations in the lake and in the Struga Biskupia (r = 0.48; *p* = 0.026), in which the P_tot_ is as high as 90 – 350 μg·L^−1^. However, from Fig. 7 it emerged that the acceleration of eutrophication coincided with the decline in the KPPP activity, which conflicts with the above conclusion because the lower KPPP activity undoubtedly translated into the lower delivery of cooling waters to LLi. Third, the increase in P_tot_ might be owing to enhanced nutrient loading from in-lake sources, primarily hypolimnion. Such conclusion is supported by statistically significant long-term increase in P_tot_ in October – December, i.e. after the breakdown of vertical stratification. This coincidence may indicate a connection between lake trophic state and a change in vertical circulation in the lake resulting from the long-term cooling. Currently, owing to the ongoing warming, the lakes tend to evolve towards more stably stratified water columns (Bartosiewicz et al., 2019). It seems likely, that owing to the ongoing cooling, LLi have followed the opposite trajectory. However, because long-term monitoring data from the water column of the lake is missing altogether, this interpretation remains hypothetic.

Many authors claim that salinization of freshwaters is usually accompanied by the increase in alkalinity because of the contribution of base salts to the latter parameter (Kaushal et al., 2018; Canedo-Argüelles 2020). Yet, in a long-term perspective, LLi has displayed a statistically significant decline in total alkalinity at a rate of 0.026 mmol·L^−1^·y^−1^ (Fig. 5). Importantly, the depletion in A_T_ in LLi was not associated with any change in pH. Despite the substantial decline, compared to natural lakes in Poland, the A_T_ values in LLi have remained exceedingly high (Table 2) implying an intense alkalinity production in the lake and/or the import of alkalinity from external sources. Given that, neither the high A_T_ producing processes (e.g. Fe/Mn oxide reduction as well as net calcium carbonate dissolution (Lerman and Stumm, 1989; Soetaert et al., 2007)) nor associated pH increase (Soetaert et al., 2007) were observed in the lake, the latter possibility seems more likely and the delivery of the high-alkalinity mining waters from the adjacent brown coal mines can be invoked as a potential A_T_ source. Indeed, during the last three decades, the SB, acting as the major conveyor of mining waters to the Konin lakes, has constantly had the A_T_ between 4.1 and 7.6 mmol·L^−1^. In addition, the long-term A_T_ trend in LLi has closely followed the temporal changes in the operability of the KPPP, which might suggest that the decommissioning of the power plants, translated into the decrease in the contribution from the SB waters in the water budget of the lake. In terms of the alkalinity model, developed by Lerman and Stumm (1989) for lakes in non-equilibrium with the atmosphere, in which

(3) [A_T_] = C_T_·(ά_1_ + 2ά_2_) + [OH^-^] + [H^+^]

**Table 2:** Alkalinity (mean ± standard deviation; mmol·L^−1^) in surface waters of Polish lakes.

Lake	Acronym	n	A_T_ ($\overset{®}{\text{x }}\pm \text{ σ})$	Source o data
			mmol·L^−1^	
**Licheńskie**	**LLi**	**102**	**4.90 ± 0.38**	**This study**
Łódzko-Dymaczewskie	LLD	13	3.32 ± 0.33	Woszczyk et al. (2023)
Szurpiły	LSz	29	3.13 ± 0.25	unp. (W. Tylmann)
Dębno	LD	13	3.04 ± 0.77	Woszczyk et al. (2023)
Kortowskie	LKor	103	3.01 ± 0.32	GIOŚ
Łazduny	LLaz	29	2.65 ± 0.32	unp. (W. Tylmann)
Trześniowskie	LT	13	2.47 ± 0.35	Woszczyk et al. (2023)
Kierskie	LKie	10	2.44 ± 0.38	Apolinarska et al. (2020)
Kamionkowskie	LKa	18	2.41 ± 0.11	GIOŚ
Sarbsko	LSar	22	2.10 ± 0.05	Woszczyk et al. (2023)
Suminko	LS	27	2.03 ± 0.28	Tylmann et al. (2012)
Jasne	LJas	15	1.69 ± 0.06	unp. (A. Pukacz)
Czarne	LCz	25	0.10 ± 0.05	GIOŚ

n – number of observations

GIOŚ – Chief Inspectorate for Environmental Protection (Poland)

unp. - unpublished

where C_T_ acts as a concentration of inorganic C species (primarily HCO_3_^-^) and ά_1_, ά_2_ are terms expressing dependence of C_T_ on water pH and temperature, under the conditions in LLi (i.e. no statistically significant change in lake water pH with a long-term average of ca 7.85 and 2.2 °C LSWT decrease), the observed decrease in alkalinity (0.714 mmol·L^−1^) is accounted for by c.a. 0.716 mmol·L^−1^ (equivalent to 44 mg·L^−1^) decline in HCO_3_^-^ over 30 years. The LSWT decrease (0.09°C/28 y) alone would have also contributed to a reduction in alkalinity albeit the effect of the observed cooling (calculated with the formula [3] for a constant pH of 7.85 and C_T_ of 4.50 and 5.00 mmol·L^−1^) was in the order of 0.01 mmol·L^−1^ of A_T_ drop, i.e. far too less than the recorded decline. Therefore, we hypothesize that the decrease in the operability of the KPPP and the resultant lowering in the delivery of cooling/mining waters to LLi is the major reason for a long-term decline in the alkalinity in LLi. On the other hand, unlike Cl^-^, the A_T_ concentrations in LLi were uncorrelated with those in the SB waters. These different patterns of A_T_ in the SB and LLi can be explained by the consumption of alkalinity in the cooling system. The consumption occurs both during water transfer from the source to the lake and within the lake itself. The former possibility is corroborated by the A_T_ gradient between the SB ad LLi, while the latter can be inferred from the annual cycle of A_T_ in LLi. As follows from Fig. S7 the consumption primarily occurs during summer and is presumably via the primary productivity and associated carbonate precipitation (Soetaert et al., 2007, Perolo et al., 2023). Persistent supersaturation of LLi waters with respect to calcite (Fig. S12) and a high carbonate content in the surface sediments of LLi (Brzozowska et al., 2007) provides evidence that intense decalcification of waters indeed occurs in the lake.

Alternative explanation for the long-term decrease in alkalinity in the lake can be ongoing eutrophication. This processes is strongly supported by the rising P_tot_ concentrations in LLi over time and the effect of this process for HCO_3_^-^ consumption was reported by Perolo et al. (2023).

Some authors claim that alkalinity in lakes is also shaped by non-carbonate components such as humic and fulvic substances. Bisogni and Arroyo (1991) acknowledged these substances in their model in the following expression

[A_T_] = [HCO_3_^-^] + 2·[CO_3_^2-^] + [OH^-^] – [H^+^] + 0.46·[A_org_]

where A_org_ acts as a function of total organic C concentration in water and pH. From this equation it follows that a decrease in A_T_ would require a decrease in organic C. Despite that the TOC/DOC in LLi have not been monitored, the long-term decline in these parameters in the lake seem very unlikely because the lake has been prone to eutrophication as well as showed constantly high values of COD, indicating the enrichment in organics in the water (Li and Liu, 2019). Even in the case that the A_org_ would decline, its effect on the A_T_ was estimated to be low because a very high (and unrealistic) change from 20 mg TOC/L to 5 mg TOC/L resulted in a 1.4 % drop in A_T_.

### 6.1 Implications for lake management

Despite being thermally polluted and subject to alkalinity-laden discharges, LLi is currently undergoing recovery from these disturbances. This shows a high capability of this system to buffer unwanted environmental changes. However, hitherto unidentified and the most persistent problem to LLi seems to be ongoing salinization. The most likely reason is saline mining water disposal to the system of Konin lakes, albeit the use of road de-icing salts and agricultural effluents may contribute to this process as reported from many regions all over the world (Herbert et al., 2015, Dugan et al., 2017, Kaushal et al., 2018, Cañedo-Argüelles, 2020, Kelly et al., 2024). Interestingly, the enhancement of salinization of the system coincided with the decrease in the activity of the KPPP albeit robust causal explanation for this coincidence is currently missing.

Unlike alkalinity, chlorides are biogeochemically inert and can hardly be removed from freshwaters via precipitation and/or sorption. Therefore, these ions can accumulate in water columns even though their delivery is declining. Provided that in the following years the Cl^-^ follow a linear increase described by the equation in Fig. 6, LLi will surpass the limit of salt pollution of 50 mg·L^−1^ (Hintz et al., 2022) by 2060. This situation is not alarming but worrisome, especially that the climate of central Poland is becoming drier (Somorowska, 2022), which may translate into enhancement of evaporation. It has been established that at higher concentrations (at least above 120 mg·L^−1^; Hintz et al., 2022) Cl^-^ have many adverse effects on the physical processes in lakes (Bielańska-Grajner and Cudak, 2014, Liu et al., 2020, Kelly et al., 2024, Ladwig et al., 2023) as well on the biota at different ecosystem levels (Dugan et al., 2017). There are reports however, that even under Cl- levels below this threshold, some ecological changes in aquatic systems have occurred (Kelly et al., 2024 and references therein). It has also been recognized that the negative biological responses to lake salinization are enhanced by decreasing water hardness because blocking of Cl^-^ penetration into the cells by Ca^2+^ and Mg^2+^ becomes less effective (Hintz et al., 2022). As the most severe transformations of LLi are more associated with the activity of the brown coal mine rather than the KPPP itself, any attempts towards restoring the lake to natural conditions or mitigating further salinization should primarily focus on the reduction of salt input. However, given that the brown coal mines in the Konin region are on a track to termination, it is possible that the salinity of LLi waters will automatically level off in the following decades, thus reducing a threat of chemical pollution to the lake. The planned NPP is not expected to deteriorate biogeochemical conditions in the lake substantially, except that the thermal pollution effect may exacerbate.

## 7 Conclusions

In the study we attempted to assess the effects of long-term use of Lake Licheńskie as a recipient of cooling waters from nearby electric power plants. This process has for many years been regarded as a major source of considerable transformations of the lake, thermal pollution in particular. In our study we showed that after 60 years of human impact, the thermal pollution in the lake primarily occurs during summer, displays a considerable spatial variability and the surface water temperatures show a long-term decreasing trend. This tendency can be seen as a recovery of the system induced by the decline in the production of electricity in the power plants. At the same time, the long-term cooling trend makes the Konin lakes an unique aquatic system which evolves oppositely to other lakes under globally warming climate.

There are however other, hitherto overlooked, environmental threats to the lake, which are more related to the delivery of mining effluents rather than cooling waters. These are alkalinization and salinization. While the former displays a declining tendency, the latter is constantly escalating, even though the mining activity in the vicinity of the lake has been reduced. Even though the rate of salinization is moderate, this unwanted effect is expected to be amplified in the following years owing to the observed increase in evaporation and the common use of salts as de-icing and anti-icing agents in Poland.

## CRediT authorship contribution statement

**Woszczyk Michal:** Writing – original draft, Investigation, Funding acquisition, Formal analysis, Data curation, Conceptualization. **Michael Brechbühler:** Writing – review & editing, Visualization, Investigation.

## Declaration of Generative AI and AI-assisted technologies in the writing process

The authors declare that any generative AI was used at any stage of the writing process.

## Declaration of Competing Interest

The authors declare the following financial interests/personal relationships which may be considered as potential competing interests: Michal Woszczyk reports financial support was provided by National Science Centre Poland. If there are other authors, they declare that they have no known competing financial interests or personal relationships that could have appeared to influence the work reported in this paper.

## Data Availability

Data will be made available on request.
