# Supplementary material for: Long term changes in the quality of the aquatic environment of thermally polluted Lake Licheńskie, Central Poland
Source: J Hydrol Reg Stud. 2025 Dec;62:102917. doi: 10.1016/j.ejrh.2025.102917 (PMC12713164; doi:10.1016/j.ejrh.2025.102917)
Supplement: Supplementary file 1 — Supplementary material [file mmc1.docx]

**Figure S1**

|  |
| --- |
| *Figure S1. The annual cycle of cooling water temperature at the inflow to the Pątnów power plant (in grey) and outflow to L. Gosławskie. The values shown represent long-term monthly means ± standard deviations. The temperature difference between inflow and outflow in on average 8.2±2.8°C with only minor seasonal variability. The chart drawn on the basis of data obtained with the courtesy of the ZEPAK SA.* |

**Figure S2**

| 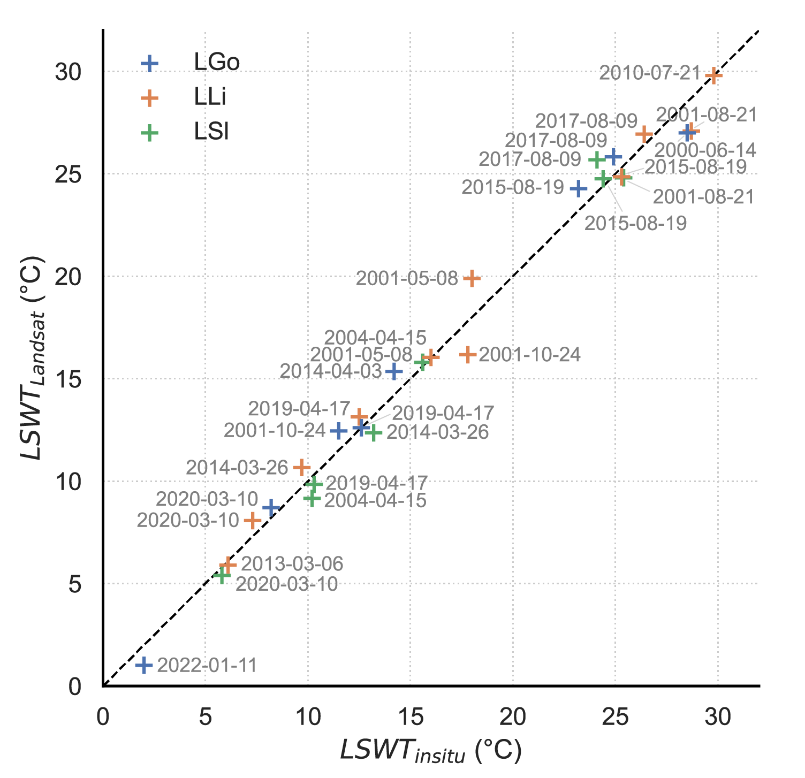 |
| --- |
| *Figure S2. Scatterplot comparing in-situ lake surface water temperature measurements (LSWT_insitu_) with corresponding satellite-derived temperatures (LSWT_Landsat_) from same-day match-ups for the Konin Lakes. Each point represents an individual in-situ-satellite observation pair. Grey text annotations indicate the date of each matchup. The 1:1 line (solid black) denotes perfect agreement between. The in-situ observations originate from historical limnological monitoring data obtained from the Department of Environmental Protection of the Zespół Elektrowni Pątnów-Adamów-Konin, Poland (ZEPAK; https://www.zepak.com.pl/en).* |

**Figure S3**

|  |
| --- |
| *Figure S3. Surface water temperatures in the Konin lakes and in L. Gopło (LGop) during 2000 – 2025. The chart shows long-term medians (white circles), 25^th^ and 75^th^ percentile (black bar) and min-max range (whiskers) of daily surface-averaged temperature values for each lake. The SWT differences between LLi and LGo are insignificant. LLi, LGo, LPa and LWM showed significantly higher SWT values than LGop.* |

**Figure S4**

| **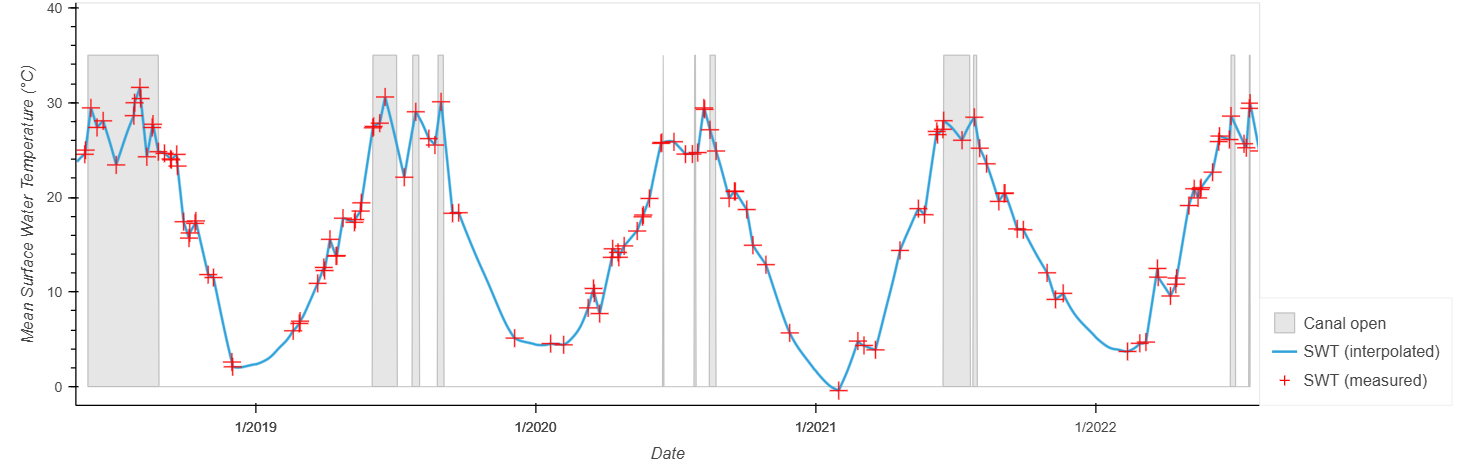** |
| --- |
| *Figure S4. The influence of the outflow to L. Ślesińskie on the SWT values in L. Licheńskie*  *The outflow is located in the north part of L. Licheńskie and opens in summer when the SWT in the lake approaches 30°C. shortly after the opening the SWT in LLi decrease sharply.* |

**Figure S5**

|  |
| --- |
| *Figure S5. Stratification stability index for Lake Licheńskie for two observation periods (December 2013 – November 2014 and March 2022 – February 2023). The Ws values increase with stratification stability. During these periods water temperature records were collected monthly at the deepest site of the lake (L1; Fig. 1) every 1 m from the water surface to the lake bottom.* |

**Figure S6**

| **A** |  |
| --- | --- |
| **B** |  |
| *Figure S6. Long term LSWT trends in Lake Gosławskie (A; closed circles, dotted line), L. Pątnowskie (A; open circles; soild line), L. Ślesińskie (B; open diamonds, solid line) and L. Wąsosko-Mikorzyńskie (B; closed diamonds; dotted line) between 2000 and 2023 inferred from spaceborne data. The p values associated with regressions equations indicate the probability that the slope is 0. The SWT in LGo and LPa show statistically significant decreasing trends (at p = 0.003 and p = 0.011, respectively), while in LWM and LSl the trends were insignificant.* | |

**Figure S7**

|  |
| --- |
| *Figure S7. The long-term annual alkalinity ranges in the surface waters of the Konin lakes between 1995 and 2023. The chart shows long-term medians (white circles), 25^th^ and 75^th^ percentile (black bar) and min-max range (whiskers) for each lake.* |

**Figure S8**

|  |
| --- |
| *Figure S8. Annual cycle of A_T_ (*$\bar{\text{x}}\text{ ± σ}$*)in L. Licheńskie on the basis of long-term data (1995 – 2023)* |

**Figure S9**

| **A** |  |
| --- | --- |
| **B** |  |
| *Figure S9. Long term A_T_ trends in Lake Gosławskie (A; closed circles, dotted line), L. Pątnowskie (A; open circles; soild line), L. Ślesińskie (B; open diamonds, solid line) and L. Wąsosko-Mikorzyńskie (B; closed diamonds; dotted line) between 2000 and 2023 inferred from spaceborne data. The p values associated with regressions equations indicate the probability that the slope is 0. The SWT in LGo, LWM and LSl show statistically significant decreasing trends (at p = 1.14·10^-5^, p = 4.7·10^-4^ and p = 1.3·10^-3^, respectively), while in LPa the trend was insignificant.* | |

**Figure S10**

|  |
| --- |
| *Figure S10. The long-term annual pH ranges in the surface waters of the Konin lakes between 1995 and 2023. The chart shows long-term medians (white circles), 25^th^ and 75^th^ percentile (black bar) and min-max range (whiskers) for each lake.* |

**Figure S11**

|  |
| --- |
| *Figure S11. Annual cycle of pH (*$\bar{\text{x}}\text{ ± min/max}$*) in L. Licheńskie on the basis of long-term data (1995 – 2023)* |

**Figure S12**

|  |
| --- |
| *Figure S12. Annual cycle of SI_calc_ (*$\bar{\text{x}}\text{ ± min/max}$*) in L. Licheńskie on the basis of long-term data (1995 – 2023)* |

**Figure S13**

|  |
| --- |
| *Figure S13. The long-term annual COD ranges in the surface waters of the Konin lakes between 1995 and 2023. The chart shows long-term medians (white circles), 25^th^ and 75^th^ percentile (black bar) and min-max range (whiskers) for each lake.* |

**Figure S14**

|  |
| --- |
| *Figure S14. The long-term annual Cl^-^ ranges in the surface waters of the Konin lakes between 1995 and 2023. The chart shows long-term medians (white circles), 25^th^ and 75^th^ percentile (black bar) and min-max range (whiskers) for each lake.* |

**Figure S15**

|  |
| --- |
| *Figure S15. Long-term changes in the concentrations of chlorides in the Konin lakes between 1995 and 2023. For all lakes the increasing trends are statistically significant. Data used with the courtesy of ZEPAK.* |

**Figure S16**

|  |
| --- |
| *Figure S16. The long-term annual P_tot_ ranges in the surface waters of the Konin lakes between 1995 and 2023. Black circles indicate mean P_tot_ and vertical bars show the range of annual means in the period studied. n is the number of values used for calculations for each lake. LLi – L. Licheńskie, LGo – L. Gosławskie, LSl – L. Ślesińskie, LWM – L. Wąsosko-Mikorzyńskie, LPat – L. Pątnowskie* |

**Figure S17**

|  |
| --- |
| *Figure S17. Annual cycle of P_tot_ (*$\bar{\text{x}}\text{ ± σ}$*) in L. Licheńskie on the basis of long-term data (1995 – 2023)* |

**Figure S18**

| A) LLi |
| --- |
|  |
| B) LLD |
|  |
| C) LD |
|  |
| D) LT |
|  |
| *Figure S18. Oxygen accumulation/consumption in the near-bottom water layer of L. Licheńskie (A), L. Łódzko-Dymaczewskie (B), L. Dębno (C) and L. Trześniowskie (D). The near bottom water layer in LLi was between 12 and 12.5 m depth (4,033 m^3^), in LLD between 11 and 12 m depth (35,067 m^3^), in LD between 10 and 11 m depth (15,333 m^3^) and in LT between 44 and 46 m depth (104,385 m^3^). Oxygen accumulation shown by black regression lines, oxygen consumption shown by red lines. Oxygen consumption rates was calculated from by dividing the slopes of the regression equations by the volume of the near bottom waters.* |

**Table S1**

*Validation statistics describing the accuracy of satellite-derived lake surface water temperature (LSWT) obtained from Landsat thermal data. The validation is based on same-day match-ups between in-situ surface water temperature measurements collected by local authorities (LSWT_insitu_) and corresponding satellite-derived temperatures (LSWT_Landsat_). The in-situ observations originate from historical limnological monitoring data obtained from the Department of Environmental Protection of the Zespół Elektrowni Pątnów-Adamów-Konin, Poland (ZEPAK; https://www.zepak.com.pl/en). For each sampling site, Landsat temperatures were extracted as the mean of a 3x3 pixel window (90x90m) centered on the measurement location. Sampling coordinates were adjusted toward the lake centerline to ensure a minimum 100 m distance from the shoreline, thereby minimizing shoreline-mixing effects associated with the native thermal sensor resolution. The table lists the number of validation points (N), mean absolute error (MAE), standard deviation of absolute errors (1 σ), standard error of the MAE (MAE SE), 95 % confidence interval of the MAE (MAE CI₉₅), root-mean-square error (RMSE), mean bias, and coefficient of determination (R²). MAE and its dispersion (MAE ± 1 σ) represent the typical magnitude and variability of differences between in-situ and satellite-derived LSWT observations.*

*LGo – L. Gosławskie, Sampling site 52°18'10.46"N 18°14'52.46"E*

*LLi – L. licheńskie, Sampling site 52°18'6.55"N 18°19'2.64"E*

*LSl – L. Ślesińskie,* *Sampling site 52°23'9.59"N 18°19'37.60"E*

| **Lake** | **N** | **MAE  [°C]** | **Error (1σ)  [°C]** | **MAE SE [°C]** | **MAE CI_95_**  **[°C]** | **RMSE**  **[°C]** | **Bias [°C]** | **R2** |
| --- | --- | --- | --- | --- | --- | --- | --- | --- |
| LGo | 8 | 0.36 | 0.41 | 0.0041 | (0.35, 0.37) | 0.55 | -0.001 | 0.96 |
| LLi | 11 | 0.39 | 0.45 | 0.0033 | (0.39, 0.4) | 0.60 | -0.009 | 0.95 |
| LSl | 9 | 0.44 | 0.56 | 0.0015 | (0.44, 0.45) | 0.71 | 0.003 | 0.93 |

**Table S2**

*Cross-validation statistics describing the reconstruction accuracy of the lake surface water temperature (LSWT) time series based on Landsat thermal data and derived using DINEOF. For each lake, 1 % of valid water pixels were withheld and subsequently reconstructed to quantify the uncertainty associated with the gap-filling step. The table lists the number of cross-validation points (N), mean absolute error (MAE), the standard deviation of absolute errors (1σ), standard error of the MAE (MAE SE), 95 % confidence interval of the MAE (MAE CI₉₅), root-mean-square error (RMSE), mean bias, and coefficient of determination (R²). MAE and its dispersion (MAE ± 1 σ) represent the typical magnitude and variability of reconstruction errors across withheld pixels.*

*LLi – L. licheńskie, LPa – L. Pątnowskie, LGo – L. Gosławskie, LSl – L. Ślesińskie, LWM – L. Wąsosko-Mikorzyńskie, LGop – L. Gopło*

| **Lake** | **N*** | **MAE** | **Error (1σ)** | **MAE SE** | **MAE CI_95_** | **RMSE** | **Bias** | **R2** |
| --- | --- | --- | --- | --- | --- | --- | --- | --- |
| Lli | 10’100 | 0.36 | 0.41 | 0.0041 | (0.35, 0.37) | 0.55 | -0.001 | 0.96 |
| LPa | 18’397 | 0.39 | 0.45 | 0.0033 | (0.39, 0.4) | 0.60 | -0.009 | 0.95 |
| LGo | 33’212 | 0.42 | 0.48 | 0.0026 | (0.42, 0.43) | 0.64 | -0.013 | 0.95 |
| LSl | 10’065 | 0.35 | 0.41 | 0.0041 | (0.34, 0.36) | 0.54 | 0.013 | 0.96 |
| LWM | 16’703 | 0.41 | 0.42 | 0.0032 | (0.4, 0.41) | 0.58 | 0.003 | 0.95 |
| LGop | 140’995 | 0.44 | 0.56 | 0.0015 | (0.44, 0.45) | 0.71 | 0.003 | 0.93 |

**Table S3**

*Statistical significance of the differences in the mean annual temperatures between the Konin lakes and Lake Gopło, a non-heated reference lake. The significance was tested with the Kruskall-Wallis test. The values reported act as probabilities (p) that the population medians compared are not different. Values in bold indicate statistically significant differences.*

*LLi – L. licheńskie, LPa – L. Pątnowskie, LGo – L. Gosławskie, LSl – L. Ślesińskie, LWM – L. Wąsosko-Mikorzyńskie, LGop – L. Gopło*

LLi LPa LWM LSl LGo LGop

LLi **0.031 0.034 2.224·10^-5^** 1 **2.613·10^-12^**

LPa 1 1 0.091 **2.609·10^-4^**

LWM 1 0.096 **2.398·10^-4^**

LSl **1.087·10^-4^** 0.160

LGo **2.885·10^-11^**

LGop

**Table S4**

*Statistical significance of the differences in the mean annual alkalinity (*A_T_*) values between the Konin lakes. The significance was tested with Kruskall-Wallis test. The values reported act as probabilities (p) that the population medians compared are not different. Values in bold indicate statistically significant differences.*

*For explanation of the acronyms see the caption for Tab. S1.*

LLi LGo LSl LWM LPa

LLi ***0.04 0.02E-01*** *0.25 0.49*

LGo ***2.05E-06 1.99E-03*** *0.32*

LSl *0.06* ***3.13E-03***

LWM *0.12*

LPa

**Table S5**

*Statistical significance of long term trend in mean monthly P_tot_ concentrations in LLi surface waters between 1995 and 2024 checked with Mann-Kendall trend test. Months with significant trends marked in bold.*

| Month | I | II | III | IV | V | VI | VII | VIII | IX | X | XI | XII |
| --- | --- | --- | --- | --- | --- | --- | --- | --- | --- | --- | --- | --- |
| S | 77 | 62 | **101** | 69 | **91** | 33 | 87 | 70 | 74 | **104** | **96** | **97** |
| Z | 1.892 | 1,521 | **2.337** | 1.235 | **1.986** | 0.633 | 1.798 | 1.367 | 1.609 | **2.410** | **2.223** | **2.244** |
| *p* (no trend) | 0.058 | 0,128 | **0.019** | 0.178 | **0.047** | 0.526 | 0.072 | 0.172 | 0.108 | **0.016** | **0.026** | **0.025** |
| Trend | NT | NT | **IT** | NT | **IT** | NT | NT | NT | NT | **IT** | **IT** | **IT** |

NT – no statistically significant trend

IT – statistically significant increasing trend
